# Rituximab and risk of COVID-19 infection and its severity in patients with MS and NMOSD

**DOI:** 10.1186/s12883-021-02218-4

**Published:** 2021-05-01

**Authors:** Sara Esmaeili, Mohammad Hossein Abbasi, Meysam Abolmaali, Mohammad Mojtahed, Seyedeh Niloufar Rafiei Alavi, Sevim Soleimani, Mahisa Mokhtari, Jaber Hatam, Samaneh Tanhapour Khotbehsara, Mohammad Reza Motamed, Mohammad Taghi Joghataei, Zahra Mirzaasgari, Mehdi Moghaddasi

**Affiliations:** 1 Cellular and Molecular Research Center, Iran university of medical sciences, Tehran, Iran; 2 School of Advanced Technologies in Medicine, Iran University of Medical Sciences, Tehran, Iran; 3 Department of Neurology, Iran University of Medical Sciences, Tehran, Iran; 4 School of Medicine, Shahid Beheshti Medical University, Tehran, Iran; 5 Department of Neurosurgery, Iran University of Medical Sciences, Tehran, Iran

**Keywords:** COVID-19, Disease modifying therapy, Multiple sclerosis, MS, Neuromyelitis Optica, NMO, Rituximab

## Abstract

**Background:**

Choosing a safe disease modifying therapy during the COVID-19 pandemic is challenging. This case series study was conducted to determine the incidence rate and the course of Covid-19 infection in MS/NMOSD patients treated with Rituximab.

**Methods:**

In this study, we designed a web-based questionnaire. Baseline information such as patient- reported walking disability, total number of Rituximab infusions received, delayed injections, occurrence of any relapse, and the use of corticosteroids during the pandemic were collected. Also, information regarding the Covid-19 pandemic such as adherence to self-isolation, any recent exposure to an infected individual and the presence of suggestive symptoms were collected. In case of positive test results, patients were grouped into 2 categories; mild to moderate and seriously ill and outcomes were evaluated as favorable (improved/ discharged) and unfavorable (expired).

**Results:**

Two hundred fifty-eight patients with Multiple Sclerosis were enrolled in this study, 9 of the subjects (3.4%) were confirmed positive for Covid-19, five of which required hospitalizations (55.5%), two patients required ICU admission (22.2%) and 2 two patients died (22.2%). None of these patients ever mentioned using corticosteroids during the pandemic. In comparison to MS patients who were not receiving disease modifying therapy (DMT), our study indicated a higher incidence of Covid-19 infection, higher ratio of serious illness and a higher fatality ratio.

**Conclusions:**

Rituximab seems not to be safe enough during the pandemic.

## Introduction

SARS-CoV-2 also known as Coronavirus disease COVID-19 has become a public health emergency of international concern [1], accounting to numerous reported neurological manifestations [2]. Smoking, older age (> 60 years old), comorbidities, and immunosuppression are associated with more severe disease [3], however, it is not clearly defined if patients with multiple sclerosis (MS) and neuromyelitis spectrum disorders (NMOSD) are at an increased risk of COVID-19 infection or at a higher risk of a more severe disease; therefore, the COVID-19 pandemic holds additional source of concern among Neurologists [4–6]. It is believed that patients with MS are at greater risk of admission in Intensive Care Unit (ICU) in case of any infection [7–9], this stems from the fact that these patients are on disease-modifying therapies (DMTs), as well as corticosteroid therapy during their attacks, which make them more immunocompromised [3]. DMTs are divided into two categories; immunomodulators and immunosuppressants [7, 10]. Evidence has demonstrated the direct or indirect role of B cells in the pathogenesis of both MS and NMO diseases [11, 12]. In this regard, Anti-CD20 monoclonal antibodies (mAbs) targeting CD20, as B-cell-depleting therapies, have been demonstrated to reduce disease activity in both diseases [13–15]. These treatment options have been suggested as current immunosuppressive disease modifying therapies (DMTs) [3, 16]. Particularly, Rituximab (RTX), a chimeric monoclonal antibody directed at CD20 positive B lymphocytes is considered a selective immunosuppressant used in different in clinical practice (500–1000 mg, every 3–6 months, or on demand at B cells detection of ≥2% of B cells) [7–10, 17]. Recent real- world experiences have shown that patients with either NMO or MS disease benefit efficiently from RTX with favorable safety and cost-effectiveness profile [17–20]. However, It has been suggested that the risk of serious infection for patients with MS or NMOSD varies depending on the DMTs being used. More efficient DMTs such as Rituximab might be associated with a higher risk of infection [8, 9] but some authorities are against this assumption [3, 21] causing uncertainties over the safety of anti-CD20 antibodies during COVID-19 pandemic [3, 21]. The incidence rate of COVID-19 infection and the course of the illness among patients using Rituximab during the outbreak is still undefined due to scarce data, while current guidelines do not provide definitive recommendations [4, 22]. Therefore, it is crucial to conduct a study to collect information in order to evaluate the clinical status of these patients and to assess the epidemiological and clinical parameters of COVID-19 patients with MS or NMOSD undergoing treatment with Rituximab. This study, along with other ongoing studies will hopefully provide us with more definitive evidences in the context of COVID-19 disease and help authorities to finally implement appropriate strategies.

## Methods

### Study population

In this analytical case series study, patients diagnosed with MS or NMOSD who were referred to the 3 hospitals in Tehran at the time the first case of COVID-19 pneumonia was reported in February 2020 until October 2020 were assessed. Patients that received Rituximab therapy as a DMT and have received at least one full dose before the COVID-19 pandemic were included in the study while patients that received the first dose of Rituximab during the pandemic were excluded.

### Study design

A web-based (online) questionnaire was used in collecting data from patients and or their caregivers. Initially, the investigators made their contact with these patients through a phone call and explained to them the purpose of the study as well as a detailed explanation on how the data will be collected. The link of the online questionnaire was then provided to the participants. Where needed, investigators were ready to help patients to fill the online-form step by step. In cases of unanswered phone calls, the researchers will try again to reach to the patient for 3 more times within the 24-h period, then, after that the patient will be considered non-responder/ missed data and was deleted from the analysis and in cases of demised patients, the corresponding caregiver will be responsible to fill-up the online questionnaire.

The online questionnaire is composed of 2 core sets of data;(1) the demographic and clinical characteristics of the disease process and (2) information related to COVID-19. Part one of the questionnaire, assessed the following parameters; age, gender, comorbidities (diabetes mellitus, hypertension, cardiovascular diseases, chronic respiratory diseases, chronic kidney diseases and malignancies), disease duration and patient-reported walking disability which was assessed with the use of the Expanded Disability Status Scale (EDSS). Also, in this part of the questionnaire, information such as; the total number of Rituximab infusions administered as well as delayed dosing schedule during the pandemic (extended interval dosing), any occurrence of relapse, relapse of symptoms and treatment plan (inpatient or outpatient) and the use of corticosteroids during the COVID-19 outbreak were collected.

The second part of the questionnaire contain questions related to COVID-19 disease that include; self-isolation, any previous close contact with COVID-19 patient and the presence of suggestive symptoms of COVID-19 infection since the start of the outbreak. Patients manifesting two of the most common symptoms of COVID-19; fever, cough, shortness of breath were considered suspicious. In addition, information regarding medical and laboratory history and their test results (positive/negative) were collected. Confirmed COVID-19 patients are those with positive RT-PCR assay of nasal and pharyngeal swab specimens or a spiral chest CT-scan indicative for COVID-19 infection as reported by a physician. Information regarding patient care management for COVID-19 positive such as hospitalization, ICU- admission or home based care and duration of hospital stay were included in this questionnaire, also, the severity of the illness which was divided into 3 categories based on the CDC guideline ^(16)^; mild to moderate (treated at home with isolation, antibiotics and hydration), severe (requires hospitalization due to dyspnea and hypoxia) and critical condition (requires ICU admission due to respiratory failure, shock or multi-organ system dysfunction) and lastly, the final outcome of the disease (recover, discharged or expired) are all contained in this questionnaire. Favorable outcome indicates patient’s recovery or discharge from the hospital while unfavorable outcome means that the patient has expired.

#### Statistical analysis

Quantitative statistics were described using means and standard deviations while qualitative data were reported by their frequencies. Case fatality ratio was calculated via the proportion of deaths from the COVID-19 disease compared to the total number of patients diagnosed with the condition.

#### Ethical approval

This study was approved by the Research Ethics Committee, Iran University of Medical Sciences (IUMS) having a Registration Code: 99–1–20-17,858.

## Results

Of the 350 patients assessed through their medical records, 312 were considered eligible and were invited by telephone call to participate in the study. Response rate among eligible individuals was (*n* = 258, 82.6%). Of these 312 patients, 168 were females (65.1%) and 90 were males (34.9%). The mean age was 41.31 ± (18–36 years old). Majority of the subjects reported no comorbidities (87.6%), 196 (75.9%) received more than 3 doses of rituximab and 184 (71.3%) expressed that scheduled doses of injection were not delayed despite the pandemic. Of the 216 (83.7%) participants who did not receive any corticosteroids during the pandemic, no relapse was reported, however, out of the 42 (16.3%) patients who received corticosteroids, 6 patients (14.3%) reported a relapse. Results are summarized in Table 1.

**Table 1:** Descriptive Analysis of demographic and clinical data

*Parameter (N)*	*Categories*	*Frequency (%)*
Gender (258)	Female	65.1%
	Male	34.9%
Comorbidities (258)	Present	12.4%
	Absent	87.6%
Disease duration (258)	< 5 years	35.7%
	5–10 years	29.4%
	> 10 years	34.9%
*Walking disability (258)*	Fully ambulatory, self-sufficient, which represents EDSS 0–3	46.8%
	Ambulatory for 500 m without aid/rest which represents EDSS 4–4.5	16.3%
	Ambulatory for 200 m without aid/rest which represents EDSS 5	14.0%
	Intermittent or unilateral constant assistance which represents EDSS 5.5–6	12.4%
	Constant bilateral assistance (canes, crutches, or braces) which represents EDSS 6.5	6.2%
	restricted to wheelchair which represents EDSS 7	4.3%
*Total Number of Rituximab injections (258)*	Once	7.8%
	Twice	16.3%
	3 times	14.7%
	> 4 times	61.2%
*Extended interval dosing (258)*	Yes	28.7%
	No	71.3%
*Relapse during the pandemic (258)*	Yes	16.3%
	No	83.7%
*Relapse management (42)*	Outpatient	35.7%
	Inpatient	9.6%
	Not treated	54.7%
*Recent corticosteroid use (42)*	Yes	14.3%
	No	85.71%
*self-isolation (258)*	Yes	87.2%
	No	12.8%
*Contact with COVID-19 Patients (258)*	Yes	4.3%
	Not Aware	95.7%
*Suspected Covid-19 infection (258)*	Yes	9.7%
	No	90.3%
*Assessment of Covid-19* *(RT-PCR or Imaging) (258)*	Performed	8.5%
	Not performed	91.5%

Based on the result of the survey, 87.2% followed self-isolation guidelines and 25 (9.6%) subjects were considered suspected cases for COVID-19 and after further evaluation, 9 (3.4%) of these patients were finally confirmed positive for corona virus. Patients are individually described on Table 2

**Table 2:** Description of the Confirmed COVID-19 cases

Case #	Age	Sex	Comorbidities	Disease Duration	***Walking Disability score***	***Rituximab injections times***	***Extended Interval Dosing***	***Recent exacerbation***	***Recent corticosteroid use***	***Self-isolation***	***COVID-19 Exposure***	***Treatment method***	***Admission duration***	***Consequence***
1	42	Male	No	13	1	Twice	No	No	No	No	Yes	Hospital admission	< 2 weeks	Death
2	59	Male	No	4	1	> 4 times	No	No	No	No	Yes	Hospital admission (ICU)	< 2 weeks	Death
3	64	Female	No	4	1	Twice	Yes	No	No	No	Yes	Home care	N/A	Cured
4	41	Male	No	12	1	> 4 times	No	No	No	Yes	No	Home care	N/A	Cured
5	46	Female	No	4	2	> 4 times	No	No	No	Yes	No	Hospital admission	< 2 weeks	Cured
6	49	Female	No	8	2	> 4 times	No	No	No	Yes	Yes	Hospital admission (ICU)	< 2 weeks	Cured
7	42	Male	No	15	2	> 4 times	No	No	No	Yes	No	Hospital admission	< 2 weeks	Cured
8	52	Female	No	6	1	Twice	No	No	No	Yes	No	Home care	N/A	Cured
9	48	Female	No	18	5	> 4 times	Yes	No	Yes	Yes	No	Home care	N/A	Cured

Patients with confirmed COVID-19 diagnosis consisted of 4 males and 5 females with mean age of 49.22 years old without any comorbidities. No recent relapses or corticosteroids use was reported by confirmed cases. Six patients had followed self-isolation and four cases reported recent contact with COVID-19 cases (Table 3). Five confirmed cases of COVID-19 were admitted to the hospital of which 2 of these patients were admitted in ICU. The rest of the patients received home-based care. Two of the patients died from COVID-19. The Mortality ratio among patients admitted to hospital was 40%.

**Table 3:** Descriptive statistics of confirmed COVID-19 cases

Parameter ((Mean ± SD/ Q1,Median,Q3) or (#\%))		Treatment strategy		
		Confirmed cases (9)	Home-based care (4)	Hospital admission (5)
Age		49.22 ± 7.94 [41–64]	51.25 ± 9.63 [41–64]	47.6 ± 7.02 [42–59]
*P*-value ^a^: 0.530 ^b^				
Sex	Male	4 (44.4%)	1 (25%)	3 (60%)
	Female	5 (55.6%)	3 (75%)	2 (40%)
Disease duration		9.33 ± 5.31 [4–18]	10.00 ± 6.32 [4–18]	8.80 ± 5.06 [4–15]
*P*-value ^a^: 0.760 ^b^				
Walking disability		1.78 ± 1.30 [1–5]	2.00 ± 2.00	1.6 ± 0.54
*P*-value ^a^: 0.730 ^c^				
Number of Rituximab injections so far	Once	0 (0%)	0 (0%)	0 (0%)
	Twice	3 (33.3%)	2 (50%)	1 (20%)
	Thrice	0 (0%)	0 (0%)	0 (0%)
	> 4 doses	6 (66.6%)	2 (50%)	4 (80%)
Extended Interval Dosing	Positive	2 (22.2%)	2 (50%)	1 (20%)
	Negative	7 (77.7%)	2 (50%)	4 (80%)
Comorbidity	Positive	0 (0%)	0 (0%)	0 (0%)
	Negative	9 (100%)	4 (100%)	5 (100%)
Recent relapse	Positive	0 (0%)	0 (0%)	0 (0%)
	Negative	9 (100%)	4 (100%)	5 (100%)
Corticosteroids consumption	positive	1 (11.1%)	1 (25%)	0 (0%)
	Negative	8 (88.8%)	3 (75%)	5 (100%)
self-isolation	Positive	6 (66.6%)	3 (75%)	3 (60%)
	Negative	3 (33.3%)	1 (25%)	2 (40%)
COVID-19 contact	Positive	4 (44.4%)	1 (25%)	4 (80%)
	Negative	5 (55.5%)	3 (75%)	1 (20%)
Final outcome	Cured/Discharged	7 (77.7%)	4 (100%)	3 (60%)
	Death	2 (22.2%)	0 (0%)	2 (40%)
Incidence Rate	Mild to Moderate Condition	Serious Condition	Case Fatality Rate	
3.4%	44.4%	11%	22.2%	

^a^: Comparison between treatment strategies which were finally required for patient’s management; ^b^: Student T-test; ^c^: Mann-Whitney U test

Among the study population, the incidence rate of COVID-19 was 3.4%. The ratio of mild to moderate illness was 44.4% and the ratio of serious COVID-19 illness was 55.5%. The ratio of favorable outcome (cured/ discharged) was 77.7% and the case fatality ratio (CFR) was 22.2% [CI: − 0.049 < *p* < 0.494] (Table 3).

Results of the study has indicated that age (*P*-value: 0.530), disease duration (*P*-value: 0.760) and walking disability score (*P*-value: 0.730) have no correlation with the type of management patients received as an indicator of severity (Table 3).

## Discussion

This is one of the first case series during the COVID-19 pandemic implying unfavorable results of using Rituximab during the pandemic.

In spite the fact that the majority of COVID-19 cases in this study experienced mild to moderate illness, the hospitalization rate (55.5%) and the proportion of patients with critical conditions (22.2%) who ended up in ICU in comparison to all confirmed COVID-19 cases are considered relatively high.

The current study has not made any comparison with other DMTs. The evidence is also scarce and controversial in this regard. However, the case fatality ratio (CFR) (22.2%) and the mortality ratio of Covid-19 among hospitalized patients (40%) in this study are much higher than those of previous reports in patients who were not receiving rituximab [23, 24]. In their own studies, Rituximab was related to an increased rate of COVID-19 infection compared to other DMTs [23, 24]. In addition, it can be noticed from a review that Anti CD-20 therapy is linked with more hospitalizations (either ventilated or not) and more deaths in patients with COVID-19 infection compared to other therapies for multiple sclerosis. However, the number of not hospitalized patients was also higher [25].

On the contrary, it has been suggested in some few reports with a similar design to ours that this drug is not linked to a greater risk of serious complications of COVID-19 illness, and they encouraged using Rituximab in MS patients [3, 8, 26, 27]^.^ But one main limitation of the mentioned studies are the small sample size, not comparing with other DMTs, and lack of accurate COVID-19 confirmation [3].

Regarding predisposing factors, the sample size is insufficient for analysis, but an overview of the medical background of the infected cases shows that the majority of these patients have been treated with Rituximab for a minimum of 2 doses on a prompt and timely manner. Females were more infected than males but the male gender experienced more severe disease and had higher mortality rate. Recent exacerbation and corticosteroids use were mostly negative and walking disability was less than 3 in most cases. Based on our findings, majority of the study population were extremely health conscious as indicated by the high percentage (96%) of patients observing strict self-isolation and promptly responded to potential symptoms of coronavirus. As noted, both patients who died of COVID-19 had not followed social distancing recommendations and had been exposed to a person with COVID-19.

To explain the results, it is believed that the T cell immunity is vital for the control of SARS-CoV-2 infection [28]. In particular, it has been shown that decrease in percentage of CD8 T-lymphocytes, and not CD4 cells, is associated with poor outcome [29]. But, the immunity against COVID-19 is complex and current studies show B-cell responses occur concomitantly in patients infected with COVID-19 disease, and most patients develop neutralizing antibodies after a delay, which are likely to be effective against SARS-CoV-2 with good clinical results [30]. Above all, some studies have demonstrated that Rituximab can also make a reduction in T-lymphocytes, especially in CD3+, CD4+, and to a lesser degree CD8+ T-cells along with a small subgroup of T-cells expressing CD20 [31]. This might justify the higher risk of serious viral infections in susceptible patients [32]. However, it is controversial whether it causes further decrease in repeated cycles of use [33] or not [34].

Regarding the general approach of using DMTs during the pandemic, recommendations are categorized into initiation, continuing, delaying, and stopping DMTs. The International Federation (MSIF) along with MS Society have recommended not to initiate Rituximab during the COVID-19 pandemic, but the Global advice for MS patients during pandemic does not suggest Rituximab discontinuation. Delaying the next doses of Rituximab should be individualized and based on patient-physician shared decision making (SDM) [35–37] In this respect, Dr. Brownlee [38] has endorsed delaying the initiation of Rituximab or considering an alternative for patients along with extended interval dosing guided by intermittent assay for CD19 lymphocyte counts for patients already on treatment. In our study, since none of the confirmed COVID-19 patients who were seriously ill had experienced delayed scheduled infusion dates, we cautiously recommend extended interval dosing if there is an immediate need for doing so.

Regarding NMOSD, discontinuation or using extended-interval dosing in these patients is discouraged, for the reason that withholding or delaying high efficacy DMTs might conversely place patients at a higher risk of relapse, which in turn results in ominous consequences. This may as well increase the chance of serious COVID-19 illness indirectly since it is hypothesized that higher EDSS scores result in less mobility and worsens the COVID-19 disease outcome [7, 37]. Altogether, thorough medical judgment including risk assessment of delaying the treatment should be decided individually and watchful surveillance is suggested.

The strong point of our study is utilization of either positive PCR or Chest CT results for verifying COVID-19 diagnosis to avoid missing cases. The limitation of this study is not comparing these patients with those being treated with other DMTs. In addition, evaluating outcomes in patients with extended interval dosing regimen is recommended in future observational studies, considering as well previous and concomitant treatments if COVID-19 is confirmed.

## Conclusion

Patients with MS or NMOSD being treated with Rituximab may be at an increased risk of COVID-19 infection and higher possibility of serious illness and mortality. The idea of extended interval dosing during the pandemic is encouraged and the important role of self-isolation and not being exposed to patients with COVID-19 is suggested.

## Data Availability

The datasets used and/or analyzed during the current study are available from the corresponding author on reasonable request.

## Abbreviations

MS: Multiple sclerosis
NMOSD: Neuromyelitis spectrum disorders
RTX: Rituximab
EDSS: Expanded Disability Status Scale
